# The Role of Sirtuins in Sarcopenia and Frailty

**DOI:** 10.14336/AD.2022.0622

**Published:** 2023-02-01

**Authors:** Masroor Anwar, Rashmita Pradhan, Sharmistha Dey, Rahul Kumar

**Affiliations:** ^1^Department of Geriatric Medicine, All India Institute of Medical Sciences, New Delhi, India; ^2^Department of Biophysics, All India Institute of Medical Sciences, New Delhi, India; ^3^Department of Biotechnology, GITAM Institute of Sciences, GITAM (Deemed to be) University, Vishakhapatnam, India

**Keywords:** Sirtuins, Frailty, Sarcopenia, Oxidative stress, Aging

## Abstract

The population of older individuals is increasing rapidly, but only a small fraction among them is able to experiences a healthy life. Due to lack of physical exercise and oxidative stress, aging leads to sarcopenia and finally end up with frailty. Sarcopenia is a component of the frailty and described as age related degenerative changes in the skeletal muscle mass, strength and quality. Though the loss of muscle strength and mass gradually seem inevitable during aging, it can be partially prevented or overcome by a deeper insight into the pathogenesis. Sirtuin protein leads to longevity across different organisms ranging from worms to mammals. Expression of sirtuin protein increases during physical exercise and thus strengthens muscle mass. Satellite cells leads to muscle repair in a SIRT1 dependent manner. In addition, SIRT1 improves insulin sensitivity and induces autophagy in the aged mice. The current paper discussed the putative role of sirtuins in sarcopenia and frailty. Moreover, it highlighted the pathways by which sirtuins can inhibit ROS production, inflammation and mitochondrial dysfunctions and therefore confers a protective role against frailty and sarcopenia. The critical role of sirtuins in the sarcopenia and frailty pathogenesis can eventually fuel the development of novel interventions by targeting sirtuins.

## Introduction

The phenomenon of aging is essentially associated with the catabolism of muscles that leads to sarcopenia and frailty. In the older population, these two syndromes have emerged as major geriatric giants, and pose a significant burden to our health care system: primarily because of high rate of multisystem decline, leading to falls, fractures, physical disability and mortality. The ICD-10-CM (M62.84) code recognizes sarcopenia as a disease and on the basis of severity, Sarcopenia in Older People by European Working Group (EWGSOP) has categorized three stages of sarcopenia; Pre-sarcopenia, which is associated with low muscle mass and normal muscle strength or physical performance while sarcopenia exhibits both low muscle mass and low muscle strength or physical performance; Severe sarcopenia, the most advanced stage, manifests itself as low muscle mass, low muscle strength, and low physical performance. Physical disability, poor quality of life and death are the detrimental effects of sarcopenia [1]. Sarcopenia finally ends up with frailty and has been identified as a crucial component of frailty in the older people and often leads to cachexia [2]. Frailty is characterised by conditions including exhaustion, weakness, and slowness, whereas sarcopenia refers to the loss of muscle mass. It is noteworthy that frailty is more prevalent in individuals exhibiting lack of physical activity and exercise [3]. Emerging evidence suggests that dietary habits and nutritional status can significantly impact the susceptibility to frailty. In particular, Mediterranean dietary pattern, regular consumption of fruits vegetables and lower consumption of processed food confers protective against the frailty [4-7].

The frail older are more susceptible to outcomes such as falls, increased impairment, hospitalization and mortality [8, 9]. Various definitions have been used to conceptualize and operationalize frailty [10, 11] and the most extensively approved was proposed by Fried *et al* in 2001 [12]. There are five characteristics of Fried’s criteria; slow motor performance, poor endurance and energy, weakness, shrinking and inadequate physical activity. An individual exhibiting 3 or more criteria out of 5 will be considered as frail.

Physical phenotype of Fried’s criteria, such as lower grip strength and slower gait speed exhibits a significant overlap with the characteristics of sarcopenia. Consequently, sarcopenia and frailty has been regarded as a common geriatric syndrome and are often manifest themselves as adverse health outcome and impaired health-related quality of life. Latest diagnostic tools like Groningen Frailty Indicator and Frailty Index of Rockwood et al, [13, 14] can well distinguish the multiple dimensions of frailty from sarcopenia. Through extensive research and a better understanding of frailty, sarcopenia has been recognized as a crucial component of the frailty [15].

Since frailty is characterised by subtle and subjective clinical features, diagnosis is often difficult, particularly during the early stage. Furthermore, a definitive therapeutic intervention is still lacking, which further highlights the requirement for a reliable biomarker. Increase in the lifespan had simultaneously led to an increase in the incidence of several age-related comorbidities and among them frailty is the most prominent. However, the mechanisms responsible for the onset of frailty are poorly understood. The current situation requires a comprehensive understanding of the underlying pathway and considering the strong association between frailty and senescence, it is imperative to explore the molecules with a strong link with senescence. The current review article describes the putative mechanistic role of an anti-senescence protein sirtuin in the pathogenesis of frailty and sarcopenia.

## Sirtuin in aging, sarcopenia, frailty

Sirtuins (silent information regulator) family consists of seven isoforms which are nicotinamide adenine dinucleotide (NAD)-dependent proteins and conserved in all domains of life. Since, last two decades, sirtuins have evolved as a critical epigenetic regulator of aging. It also mediates the consequences of calorie restriction (CR), the only dietary intervention that deaccelerates the process of aging and extends lifespan [16]. Moreover, the beneficial effects of CR get abrogated in global SIRT1 knockout [17] and brain-specific knockout mice [18]. In addition, SIRT5 and SIRT6 overexpress in the animals fed on CR diet [19, 20]. Moreover, SIRT6 overexpression in transgenic mice leads to lifespan extension. SIRT3 also mediates the effect of CR *in vivo* [21] and gained particular interest due to its localization in the mitochondria and associated with longevity in humans [22]. SIRT1’s role in CR was validated by a clinical study which reported its overexpression in the individuals fed on a CR diet [23]. Furthermore, a previous study made an interesting observation that the expression level SIRT1 and SIRT3 in serum downregulates with age [24, 25].

NAD^+^, which acts as a cofactor for several vital enzymes like Poly (ADP-ribose) polymerase (PARP), sirtuins, and CD38, decreases with sarcopenia [26]. A reduction in their enzymatic activity impairs mitochondrial function and decreases the muscle strength [27]. Deacetylation of peroxisome proliferator-activated receptor coactivator 1-α (PGC1α) by SIRT1 *in vitro* as well as *in vivo* lead to the stabilization of mitochondria in skeletal muscle [28]. During aging, satellite cells plays a vital role in the muscle repair via SIRT1 dependent manner [29]. Several *in vivo* studies suggests that sarcopenia is characterized by a decrease in the activity and expression of SIRT1 [30-32]. SIRT1 activity also decreases with aging *in vivo*, which causes PARP-1 hyper-acetylation and NAD^+^ decrease consequently, which further inhibits activity of SIRT1. PARP-1 acetylation also leads to the stimulation of NF-κB dependent gene expression [32], which leads to increase in inflammation, one of the hallmarks of sarcopenia.

The role of sirtuins in frailty for the first time was determined by Le Couteur *et al* in 2010 in their landmark study which concluded that there was no significant difference in the level of induced SIRT1 in SK Hep1 cells upon treatment with serum collected from frail and non-frail individuals [33]. Authors also stated the possible existence of reverse association between lower SIRT1 level and the robustness. In a clinical study, Kumar et al. determined the level of different sirtuin in the serum and observed that expression of SIRT1 and SIRT3 decreases with frailty [34].

Surprisingly, Ma *et al* reported that a higher level of SIRT1 is present in frail individuals, an observation that was in contradiction to the previous study [35]. Another study observed that no significant association exists between SIRT1 single nucleotide polymorphisms (SNPs) and frail status. However, they detected the presence of a weak association between SNPs and conditions such as arthritis, cognitive impairment, and hearing impairment [36]. Overexpression of SIRT6 *in vivo* can reverse the age-associated decline in physical activity and prevents the onset of frailty at old age [37]. These studies indicate the possible mechanistic association between sirtuins and frailty, although the results are contradictory. Therefore, further studies in multiple cohorts are essential to address these contradictions.

## Mechanisms

**Figure 1.**
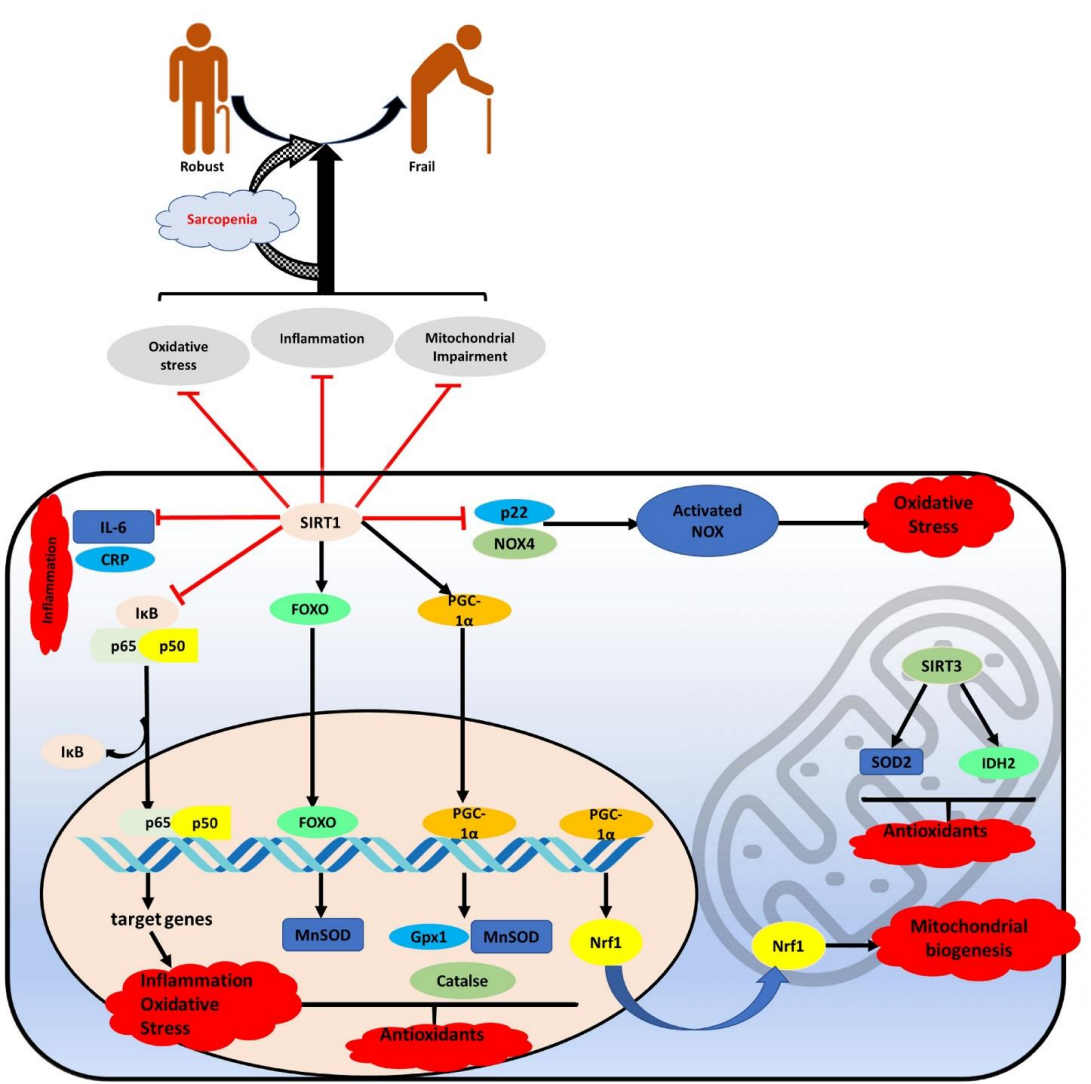
Mechanisms by which SIRT1 and SIRT3 can influence the pathophysiology of sarcopenia.

In the previous section, we described the possible association between sirtuins and sarcopenia/frailty, although the mechanisms are still elusive. Based on the currently available evidence, we suspect the following mechanisms are responsible in driving the effects of sirtuins in sarcopenia as well as frailty. Figure 1 illustrated the mechanism suspect of different pathways of SIRT1 and SIRT3 in pathophysiology of sarcopenia that ends up with frailty.

### Oxidative stress:

Several *in vivo* studies suggests that oxidative stress plays a key role in the induction of sarcopenia in different experimental models [38, 39]. In aged mice, over-expression of an enzyme glucose-6-phosphate dehydrogenase (G6PD), responsible for reducing the oxidative stress, improved neuromuscular performance [40]. Emerging clinical suggests that the level of oxidative stress is significantly higher in frail individuals in comparison to non-frail [41, 42]. Mice that lacks antioxidant Cu/Zn superoxide dismutase (SOD) exhibits sarcopenia [43]. SIRT1 deacetylate and activate FOXO3A *in vitro*, which enhances transcription of manganese SOD (Mn-SOD) [44, 45]. SIRT1 deacetylate and activates PGC-1α which enhances the expression of antioxidant likes MnSOD, catalase and glutathione peroxidase (GPx1) [28, 46-48]. Moreover, inhibition of SIRT1 leads to the overexpression of NADPH oxidase (NOX) subunits p22phox and NOX4 and increases the level of ROS production [49]. Deacetylation and activation of endothelial nitric oxide synthase (eNOs) by SIRT1 augment the NO production, which acts as a potential antioxidant [50]. Mounting evidence suggests that SIRT3, a mitochondrial sirtuin, plays a vital role in preventing ROS formation via different mechanisms. It directly deacetylates SOD-2 at two lysine residues and enhances its activity [51-53]. Reduced glutathione, a potent antioxidant compound, generates from oxidized glutathione in a reaction that requires NADPH and thereby validates the role of SIRT3 as an antioxidant molecule [21].

### Inflammation:

The first experimental association between frailty and inflammation was well-established by Leng and colleagues, who observed the presence of a higher expression of serum interleukin 6 (IL-6) in frail individuals [54]. Another clinical study indicated that frailty is characterized by an increased level of a C-reactive protein (CRP) and IL-6 [55-57]. Additional inflammatory markers like C-X-C motif chemokine ligand 10 (CXCL10) and neopterin also increases with frailty as per different clinical reports [58-60]. In addition, increase in the levels of IL-6 and CRP enhances the possible risk of sarcopenia [61]. Altogether, these studies point towards the crucial role of inflammation in the onset of sarcopenia and frailty.

### Mitochondrial dysfunction:

Emerging evidence suggests that mitochondrial dysfunction leads to sarcopenia and frailty. For example, Andreux *et al* reported a decrease in the level of proteins involved in the mitochondrial respiratory complex and an impaired phosphocreatine recovery in pre-frail individuals [62]. SIRT1 dependent deacetylation and activation of PGC-1α is a crucial pathway in the biogenesis of mitochondria [47, 63-65]. Activated PGC-1α stimulate the expression of Nuclear Respiratory Factor 1/2 (NRF1/NRF2) and transcription factor A, mitochondrial (TFAM), an essential step in mitochondrial biogenesis [66]. SIRT3 deacetylates several mitochondrial enzymes and regulates ATP production [67].

### Sirtuin as a marker/therapeutic target

Sirtuins gained significant momentum recently, based on several studies which revealed their potential as a therapeutic target and biomarker. Notably, serum sirtuins can be proposed as a promising marker for sarcopenia and frailty but multiple cohort-based studies are warranted to establish the fact. SIRT1 and SIRT3 [68] displayed a stronger association with frailty and possess the potential to be used as biomarker to prevent the progression to bed-bound phase by detect frailty at an early stage. However, future studies with a larger sample size in multiple cohorts is required to ascertain the role of sirtuins as a marker for the disease onset. Regular exercise and nutritional status had emerged as an essential intervention to prolong lifespan and increase muscle mass. Resveratrol, the activator of SIRT1 improves the effectiveness of exercise on the satellite cell activation in older individuals [46]. A previous study had shown a significant improvement in the state of sarcopenia by the effectiveness of physical activity on mitochondrial enzymes as well as muscle stem cells [69]. Resistance exercise improved muscle strength and mass and proved to be effective in reversing the status of sarcopenia [70-72]. Acute exercise activates SIRT1 and not SIRT3, via phosphorylation of AMPK. Moreover, several sessions of exercise training can lead to activation of both SIRT1 and SIRT3, together with the improvement in mitochondrial oxidative function and biogenesis [73, 74].

Resveratrol can prevent the tumour necrosis factor alpha (TNF-α) induced muscle cells atrophy by restoring Akt/mTOR/S6K and 4E-BP1 signaling *in vivo* [75]. SRT2104 dependent SIRT1 activation can alleviate the loss of muscle mass in mice [76]. Exercise and resveratrol inhibit age-related changes in the gastrocnemius muscle in mice, via activation of SIRT1, PGC-1 α and 5'AMP-activated protein kinase (AMPK) [77, 78]. Resveratrol improves the forelimb grip strength in aged rats and confers protection to the cultured cells against peroxides [79, 80]. By activating SIRT1, myricanol alleviates dexamethasone-induced skeletal muscle wasting and weakness, which in turn enhances autophagy and promotes mitochondrial biogenesis [81]. Moreover, inhibition of SIRT1 is necessary for Toll-like receptor 9 (TLR9) dependent muscle fibrosis and sarcopenia in aged mice [82]. Resveratrol also protects the mice against negative health consequences of a high-fat diet. Juzentaihoto, a Chinese herbal medicine, prevents muscle atrophy in senescence accelerated mouse (SAMP8) via activation of SIRT1 [83]. Bring together, all these findings suggest that SIRT1 activation plays a pivotal role in the protection against age associated sarcopenia. However, the exact role of sirtuins in frailty is still elusive due to the lack of reliable animal models. Therefore, future studies aimed to develop an appropriate animal model of frailty to identify the exact mechanistic contribution of sirtuin in frailty and exploit them as a therapeutic target. Further longitudinal studies with frail aged individual having several age-related diseases like cognitive impairment, hypertension, diabetes etc are required for future studies

## Conclusions

The current review summarized the putative role of sirtuins in sarcopenia and frailty pathogenesis in the older people. This review highlighted the pathways by which sirtuins can influence ROS production, inflammation and mitochondrial dysfunctions to exhibit a protective role against frailty and sarcopenia in the older and its therapeutic intervention in the future. However, a reliable biomarker and efficient therapeutic interventions is still not available for frailty. Sirtuins have unique features including its different complex catalytic mechanism and substrate specificities, which offer great opportunities for the development of drug. Several pharmacological and natural activators of sirtuins, particularly SIRT1 have been under investigation for long and have shown promising results. Although there are promising *in vitro* studies with convincing results, its potential as therapeutic intervention *in vivo* and clinical studies remains completely unknown. The previous reports suggests that sirtuins plays a protective role during the onset of frailty by preventing ROS accumulation, inflammation and mitochondrial impairment. However, it is certain that novel modulators targeting SIRT1 and SIRT3 will be explored in the near future, which requires further unravelling the molecular pathway involved in frailty and its component sarcopenia. Moreover, Sirtuins can serve as potential biomarker for early intervention and combat frailty and sarcopenia therapeutically. Based on the previous literature, we suggest that SIRT1 plays a protective role during frailty and its activation can provide a novel therapeutic approach. Moreover, future studies directed towards examining the role of SIRT1 as an early marker for frailty can provide us with an approach to arrest the progression into advanced stages.
